# Beetroot (*Beta vulgaris*) rescues mice from γ-ray irradiation by accelerating hematopoiesis and curtailing immunosuppression

**DOI:** 10.1080/13880209.2016.1237976

**Published:** 2016-12-07

**Authors:** Jinhee Cho, So Jin Bing, Areum Kim, Nam Ho Lee, Sang-Hee Byeon, Gi-Ok Kim, Youngheun Jee

**Affiliations:** a Department of Veterinary Medicine and Veterinary Medical Research Institute, Jeju National University, Jeju, Republic of Korea;; b Department of Advanced Convergence Technology & Science, Jeju National University, Jeju, Republic of Korea;; c Department of Chemistry, Jeju National University, Jeju, Republic of Korea;; d Jeju Diversity Research Institute, Seogwipo, Republic of Korea

**Keywords:** Ionizing radiation, splenocytes, bone marrow cells, radioprotective effects

## Abstract

**Context:** Beetroot [*Beta vulgaris* Linné (Chenopodiaceae)], a vegetable usually consumed as a food or a medicinal plant in Europe, has been reported to have antioxidant and anti-inflammatory properties. Since the lymphohematopoietic system is the most sensitive tissue to ionizing radiation, protecting it from radiation damage is one of the best ways to decrease detrimental effects from radiation exposure.

**Objective:** In this study, we evaluated the radio-protective effects of beetroot in hematopoietic stem cells (HSCs) and progenitor cells.

**Materials and methods:** Beetroot extract was administered at a dose of 400 mg/mouse *per os* (p.o.) three times into C57BL/6 mice and, at day 10 after γ-ray irradiation, diverse molecular presentations were measured and compared against non-irradiated and irradiated mice with PBS treatments. Survival of beetroot-fed and unfed irradiated animal was also compared.

**Results:** Beetroot not only stimulated cell proliferation, but also minimized DNA damage of splenocytes. Beetroot also repopulated S-phase cells and increased Ki-67 or c-Kit positive cells in bone marrow. Moreover, beetroot-treated mice showed notable boosting of differentiation of HSCs into burst-forming units-erythroid along with increased production of IL-3. Also, beetroot-treated mice displayed enhancement in the level of hematocrit and hemoglobin as well as the number of red blood cell in peripheral blood. Beetroot diet improved survival rate of lethally exposed mice with a dose reduction factor (DRF) of 1.1.

**Discussion and conclusion:** These results suggest that beetroot has the potency to preserve bone marrow integrity and stimulate the differentiation of HSCs against ionizing radiation.

## Introduction

Radiation, particularly the ionizing radiation, has been reported to cause oxidative damage to DNA through free radicals or reactive oxygen species (ROS) (Ewing & Jones 1987). High-level exposure to ionizing radiation causes changes in the gene expression pattern, mutation prevalence and the weakening of DNA damage repair mechanisms (Cramers et al. 2011; Mukherjee et al. 2012). Hematopoietic stem cells (HSCs) and immune cells are especially vulnerable and, ensuing irradiation, the immune suppression occurs as an undesirable outcome (Harrington et al. 1997). Also, exposure to ionizing radiation causes severe depletion of progenitor cells in the bone marrow and induces the hematopoietic syndrome (Heylmann et al. 2014). As a consequence, radioprotectors are essential in both planned exposures like radiotherapy and unplanned exposures like natural background radiation alike for controlling detrimental effects (Bump & Malaker 1997; Nair et al. 2001). Despite synthetic compounds such as thiols, baminothiols, thiadiazoles or benzothiazoles that are reported to have radioprotective effects, they all have unwanted side effects (Copp et al. 2013) and the focus has been moved to the development of natural radioprotectors from plants and herbs (Citrin et al. 2010; Pal et al. 2013). Recently, natural plant extracts and plant-derived compounds such as genistein or ginsan have been shown to have the ability to modulate radiation-induced damage (Regenbrecht et al. 2008; Park et al. 2011; Bing et al. 2014).

Beetroot [*Beta vulgaris* Linné (Chenopodiaceae)], or garden beet, with red, magenta or white body and small green leaves with thin red veins is mainly cultivated for food, food colouring, or medicine in Europe, and various reports indicated its potential anti-inflammatory and antioxidant activities (for general review, see Ninfali & Angelino 2013). For example, aqueous extracts of beetroot demonstrated the anti-inflammatory activity in carrageenan-induced rat paw oedema model and in cotton pellet-induced granuloma rat model (Jain et al. 2011); ethanol extracts of beetroot roots showed anti-inflammatory effects against both xylene-induced ear oedema and cotton pellet-induced granuloma in rats (Atta & Alokfahi 1998); beetroot pomace showed the antiradical activity towards DPPH and hydroxyl radicals (Vulic et al. 2013); beetroot juice protected male Wistar rats from oxidative stress induced by carbon tetrachloride (CCl_4_) and reduced plasma protein carbonyls and DNA damage in blood leukocytes (Kujawska et al. 2009).

Beetroot contains a large amount of pigments such as betaxanthins and betacyanin of the betalain family, a group of water-soluble nitrogen containing pigments derived from betalamic acid and most studies indicate betalains as health protective molecules in beetroots. Betalains are related to anti-oxidative stress, anti-inflammation and antitumor effects of beetroots (Ninfali & Angelino 2013); both betaxanthins and betacyanins extracted from beetroot were demonstrated to have anti-radical effect when measured by the loss of 2,2′-azino-bis(3-ethylbenzthiazoline-6-sulphonic acid) radical (ABTS) (Escribano et al. 1998); betanin, the major betacyanin pigment of beetroot, has been reported to provide a strong anti-inflammatory activity by inhibiting of cyclooxygenase (COX) family and by scavenging hypochlorous acid, oxidants produced by neutrophils, during the inflammation (Reddy et al. 2005; Allegraa et al. 2005). Furthermore, betalains from cactus pear fruits were taken up by human red blood cells and protected them from oxidative hemolysis (Tesoriere et al. 2005).

Intriguingly, despite such a well-documented association to anti-inflammatory, antioxidative stress effects, the radioprotective capacity of beetroots has not been studied much. Hypothesizing that some extracts of Beetroot may protect radiosensitive cells of mice from damages induced by irradiation, we tried to determine the protective effect of beetroot against γ-ray induced damages in immune cells and HSCs of C57BL/6 mice. We demonstrate in this study that beetroot has the potency against radiation-induced damage and it has the possibility as a radioprotective agent.

## Materials and methods

### Preparation of beetroot (beta vulgaris) extracts

Freeze-dried beetroot (996.0 g) was pulverized into powder, and extracted with 70% ethanol (20.0 L) at room temperature for 24 h. The mixture was filtered, and the solution was concentrated by using rotary evaporator with the bath temperature lower than 40 °C to afford a gummy extract (105.7 g, 10.6% yield). A part of the extract (67.5 g) was suspended over distilled water (3.0 L), and partitioned successively to give fractions of *n*-hexane (0.1 g), ethyl acetate (0.4 g) and *n*-butanol (7.8 g). Most of the biomass was collected in the residual water fraction (55.6 g).

### Animals

C57BL/6 mice (Orientbio, Sungnam, Korea) were housed in conventional animal facilities with an NIH-07-approved diet and water *ad libitum* at a constant temperature (23 ± 3 °C) and humidity (50 ± 5%) according to the guidelines for the Care and Use of Laboratory Animals of the institutional Ethical Committee of Jeju National University. Mice were 24–30 g of weight and 10–15 weeks of age. Mice were randomly separated into three groups (3–4 mice/group): non-irradiated group (Naive), irradiated control group (IR) and irradiation plus beetroot-treated group (IR + Beetroot). Experiments were repeated three times with a minimum of three mice in each.

### Irradiation with ^60^Co-γ-ray

A ^60^Co irradiator (Theratron-780 teletherapy unit, Applied Radiological Science Institute, Jeju National University, Korea) was used to irradiate mice and splenocytes. Briefly, each mouse was situated in a close-fitting Perspex box (3 × 3 × 11 cm) and received 7 Gy WBI at a dose rate of 0.69 Gy/min. For *in vitro* assay, splenocytes were exposed to 1.5 Gy.

### Treatment with beetroot

Beetroot extract dissolved in phosphate-buffered saline phosphate-buffered saline (PBS; 137 mM NaCl, 2.7 mM KCl, 10 mM Na_2_HPO_4_, 1.8 mM KH_2_PO_4_, pH 7.4) was used at a dose of 400 mg/mouse p.o. into the mice in IR + Beetroot group. Beetroot extract was dissolved in PBS at a concentration of 1 g/mL and administered with 0.4 mL of the stock solution. Each mouse in IR + Beetroot group was injected three times, first at 17 h and then at 1 h before irradiation (Bing et al. 2014), and then 5 days after irradiation. For comparison, naive and IR groups were administered with the same volume of PBS only.

### Preparation of primary splenocytes

For *in vitro* assay, splenocytes were isolated from C57BL/6 mice and exposed to 1.5 Gy of γ-ray irradiation. Aliquots of splenocyte suspension were placed in a 96-well microtiter plate (Nunc, Copenhagen, Denmark) and splenocytes treated with various concentrations of beetroot extract were cultured at 37 °C, 5% CO_2_.

For *in vivo* assay, spleens were removed from mice of each group 10 days after an exposure to 7 Gy WBI. Single-cell suspensions were obtained using a cell strainer. They were treated with ammonium chloride-potassium (ACK) buffer for 10 min to lyse erythrocytes and washed with Dulbecco’s phosphate-buffered saline (DPBS, Gibco-BRL, Paisely, UK). Then, splenocytes were suspended in RPMI-1640 (Gibco-BRL) with 10% fetal bovine serum (Gibco-BRL) and 100 U/mL penicillin-streptomycin (Gibco-BRL). Cell viability was determined by Trypan blue dye exclusion (Sigma-Aldrich, MO) and the purified cells were put to use for experiments.

### MTT assay

The viability of non-irradiated or irradiated splenocytes was measured by using MTT (3-(4,5-dimethylthiazol-2-yl)-2,5-diphenyltetrazolium bromide) assay. Splenocytes were seeded onto 96-well microtiter plates at a density of 1 × 10^5^ cells/well and incubated in the presence of beetroot extract at various concentrations (0–1000 μg/mL). After incubation for 68 h at 37 °C under 5% CO_2_, 15 μL of MTT solution (5 mg/mL, Sigma-Aldrich) was added to each well to react for 4 h. Formazan crystals were dissolved in 100 μL of solubilization buffer (pH 4.7) containing 50% dimethylformamide (DMSO, Sigma-Aldrich) and 10% sodium dodecyl sulfate (SDS, Sigma-Aldrich), and the absorbance was measured at 570 nm and 630 nm by an enzyme-linked immunosorbent assay (ELISA) plate reader.

### ^3^H-Thymidine incorporation assay

^3^H-Thymidine incorporation assay was performed on irradiated splenocytes or splenocytes of experimental mice to determine whether beetroot extract stimulated the proliferation of splenocytes inhibited by irradiation. Splenocytes were seeded at a density of 5 × 10^5^ cells/well onto 96-well microtiter plates (Nunc) in triplicate and then cultured at 37 °C under 5% CO_2_. 1 μC_i_ of ^3^H-thymidine (specific activity 42 C_i_/mmol, Amersham, Arlington Heights, IL) was added to each well after 54 h incubation. After 18 h incubation, cells were harvested onto glass fibre filters by using an automatic cell harvester, and the amount of radioactivity incorporated into DNA was determined by liquid scintillation spectrometer (Wallac Micro Beta® TriLux, Perkiin Elmer, Waltham, MA).

### Alkaline comet assay

To determine whether the beetroot extract protected splenocytes from DNA damage induced by γ-ray irradiation, the alkaline comet assay was performed. Splenocytes were isolated from mice of each group 10 days after 7 Gy WBI. The alkaline technique was performed as described previously (Park et al. 2008). The percentage of comet tail length, olive tail movement and intensity of tail DNA of 100 cells/slide were analyzed using Komet 5.5 software (Kinetic Imaging Ltd., U.K.) under a fluorescence microscope.

### Tissue processing and haematoxylin and eosin (H&E) staining

Femurs of each mouse were collected 10 days after 7 Gy WBI to determine whether beetroot extract could repopulate hemopoietic stem cells in irradiated mice. Isolated femurs were fixed in 20% neutral-buffered formalin (Junsei, Tokyo, Japan) and decalcified in 10% neutral-buffered formalin containing 3% nitrate (ICN Biomedicals Inc., Costa Mesa, CA) for one week. Decalcified femurs were embedded in paraffin to obtain 3 μm paraffin sections. Haematoxylin and eosin (H&E) staining was performed to examine the cellularity of femur bone marrow. Slides were analyzed under a light microscope (Leica, New York, NY).

### Preparation of bone marrow cell suspensions

Bone marrow cells were harvested from both femurs of each mouse 10 days after 7 Gy WBI. Femur bone marrow cells were flushed out with PBS and single-cell suspensions were obtained using cell strainer. After the lysis of red blood cells, bone marrow cells were suspended in RPMI-1640 (Gibco-BRL) with 10% fetal bovine serum (Gibco-BRL) and 100 U/mL antibiotics (Gibco-BRL).

### Bone marrow cell counting

Bone marrow cell counting was performed to determine whether the beetroot extract protected bone marrow cells from depletion induced by γ-ray irradiation. Bone marrow cells from mice of each experiment group (Naïve, IR and IR + Beetroot group) were isolated 10 days after WBI, and then counted in a hemacytometer (Superior, Marienfeld, Germany) under an optical microscope.

### Cell cycle analysis by propidium iodide (PI) staining

To determine whether beetroot extract affected the cell cycle of bone marrow cells of irradiated mice, PI staining was performed. Bone marrow cells isolated from mice of each experiment group were collected and fixed with 70% EtOH at 10 days after 7 Gy WBI. Then, 1 × 10^6^ cells were resuspended in propidium iodide (20 μg/mL, Sigma) and RNase A (200 μg/mL Sigma), and then incubated at 37 °C under 5% CO_2_. After 30 min, samples were analyzed by assessing the proportion of sub G1 cells using a BD FACS Calibur^TM^ flow cytometer (BD Biosciences, San Jose, CA).

### Immunohistochemistry

For the detection of immunoreactivity of Ki-67 and c-kit, sections were deparaffinized and rehydrated using standard methods. Slides were immersed in the 0.3% H_2_O_2_ solutions to block endogenous peroxidase and then incubated with normal goat serum to block nonspecific bindings. Then sections were reacted with rabbit anti-mouse Ki-67 antibody (1:200; Abcam, Cambrige, UK) or rabbit anti-mouse c-kit antibody (1:400; Cell Signaling Technology, Inc., Danvers, MA) overnight at 4 °C. Biotinylated goat anti-rabbit IgG followed by avidin-biotin-peroxidase complexes (ABC complexes; Vector Laboratories, Burlingame, CA) were used as secondary antibodies. 3,3′-diaminbenzidine tetrachloride (DAB; Vector Laboratories) was used for detecting horseradish peroxidase (HRP) binding sites and counterstained with 2.5% Mayer’s hematoxylin (Sigma-Aldrich) for 1 min. Slides were then mounted with Canada balsam (Junsei) and analyzed with light microscope (LeicaDM LB2, Leica, Wetzler, Germany). Positive cells stained by immunohistochemistry were quantified using Image J 1.48v software (National Institutes of Health (NIH), Bethesda, MD).

### Colony forming cell (CFC) assay

Colony forming cell (CFC) assay was performed on marrow cells of experimental mice to evaluate whether the beetroot extract stimulated the proliferation of marrow cells inhibited by 7 Gy WBI. Marrow cell suspensions were seeded at a density of 5 × 10^4^ cells/well onto 6-well plates (Nunc) with methylcellulose-based media (R&D systems, Minneapolis, MN) for 12 days at 37 °C under 5% CO_2_. After incubation, colonies of burst forming unit-erythroid (BFU-E), colony forming unit-granulocyte-macrophage (CFU-GM), colony forming unit-granulocyte (CFU-G), colony forming unit-macrophage (CFU-M) and colony forming unit-granulocyte-erythrocyte-macrophage-megakaryocyte (CFU-GEMM) were counted according to manufacturer’s instructions.

### Enzyme-linked immunosorbent assay (ELISA)

To detect the effect of the beetroot extract on the production of hematopoietic cytokines, ELISA was performed. Splenocytes or marrow cells isolated from mice 10 days after 7 Gy irradiation were seeded at 1 × 10^6^ cells/well onto 96-well plates (Nunc) and then incubated at 37 °C under 5% CO_2_. After 48 h incubation, the levels of GM-CSF and IL-3 were measured in culture supernatants by using ELISA Kits (Invitrogen, Waltham, CA) according to manufacturer’s instructions.

### Peripheral blood analysis

Peripheral blood analysis was performed to elucidate the effect of the extract of beetroot on peripheral blood of mice of each experiment group. Whole blood of each mouse was collected in ethylene-diaminetetraacetic acid dipotassium (EDTA-2K)-coated blood collection tubes (BD Microtainer^®^, BD Biosciences) by cardiac puncture at 10 days after 7 Gy WBI. Levels of red blood cell (RBC, 10^6^/μL), white blood cell (WBC, 10^3^/μL), haemoglobin (HGB, g/dL), haematocrit (HCT, %), mean corpuscular volume (HCV, fl), mean corpuscular haemoglobin (MCH, pg), mean corpuscular haemoglobin concentration (MCHC, g/dL), platelet count (PLT, ×10^3^/μL) were measured by a blood cell counter (pocH-100iv, Sysmex corporation, Tokyo, Japan).

### Animal survival assessments

To evaluate the radioprotective capacity of beetroot extract, animals from two experiment groups (IR and IR + Beetroot groups) were monitored daily for survival until 30 days after exposure to 8 or 9 Gy of γ-ray irradiation. For this determination, beetroot extract-containing murine diet pellets were prepared by mixing beetroot extracts and NIH-07-approved diet in the ratio of three to seven (Dr. Seong, Korea Food Research Institute, Gyeonggi-do, Republic of Korea) and supplied to mice in IR + Beetroot group *ad libitum*. Naive and IR group received NIH-07-approved diet for comparison. They all had free access to water. Administration of their respective diets started 14 days before WBI and continued for 14 days after irradiation. Surviving mice were euthanized by cervical dislocation at 31 days after WBI.

### Statistical analysis

Results are reported as means ± the standard error of the mean (SEM). Statistical significance was analyzed by one-way ANOVA followed by Turkey’s HSD test. For the survival test, the Mann–Whitney test was performed for statistical analysis. A value of *p* < .05 was considered statistically significant.

## Results

### Beetroot extract is noncytotoxic to primary murine splenocytes

**Figure 1.**
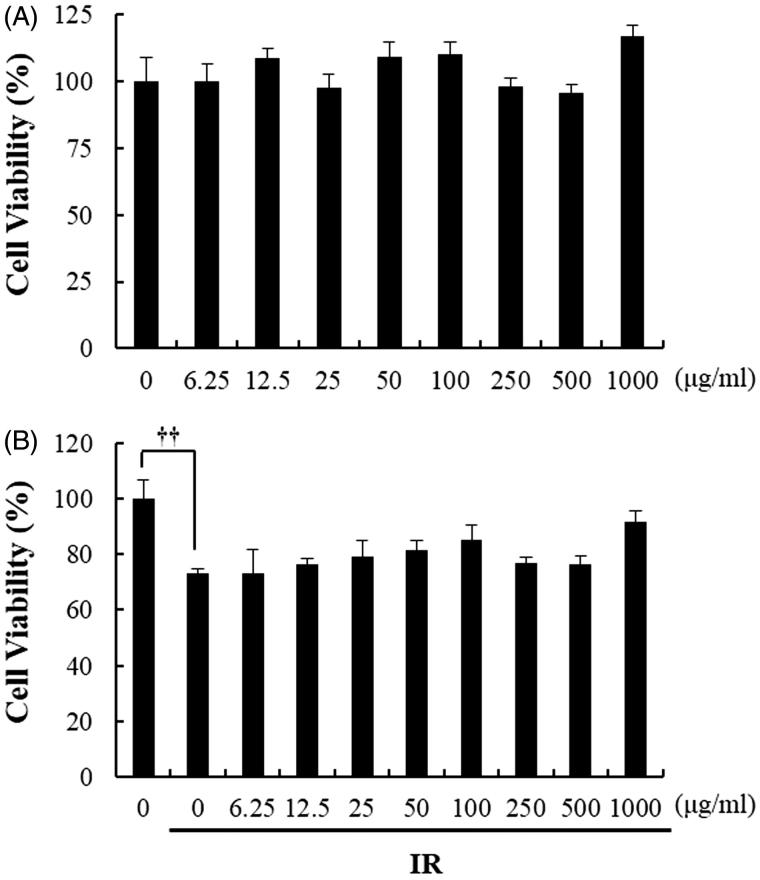
The effect of beetroot on the viability of non-irradiated or irradiated splenocytes. Cell viability of splenocytes treated with beetroot extract at various concentrations (0–1000 μg/mL) and incubated for 72 hours was measured by using the MTT assay. (a) Non-irradiated splenocytes, (b) splenocytes irradiated with 1.5 Gy γ-rays (††*p <* .01).

Before confirming the radioprotective effect of beetroot extract in cultured splenocytes, we first performed MTT assay to evaluate whether beetroot extract was cytotoxic to splenocytes. As shown in Figure 1, beetroot extract was not cytotoxic in non-irradiated splenocytes and irradiated splenocytes treated with beetroot extracts of concentration from 6.25 to 1000 μg/mL.

### Beetroot extract promotes the proliferation ability of irradiated cultured splenocytes

**Figure 2.**
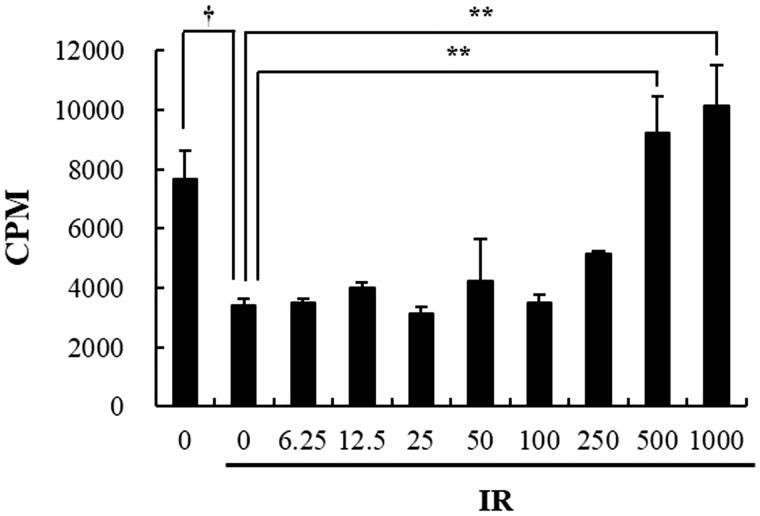
The effect of beetroot on the proliferation of irradiated splenocytes. Splenocytes irradiated with 1.5 Gy γ-ray irradiation were cultured in the presence of beetroot extract at various concentrations (0–1000 μg/mL) for 72 h and cell proliferation was measured using the ^3^H-thymidine incorporation assay. Data shown are representative of three independent experiments. Proliferative responses were assessed in triplicate for each experiment. Data are represented as means ± SEM of radioactivity-count per minute (cpm) (†*p* < .05, **p < .01).

Next, ^3^H-thymidine incorporation assay was performed to determine whether beetroot stimulated the proliferation of splenocytes after γ-ray irradiation. Proliferation levels of non-irradiated splenocytes, irradiated control splenocytes, irradiated beetroot-treated splenocytes (500 and 1000 μg/mL) or non-irradiated concanavalin A (ConA)-treated splenocytes measured by incorporation of ^3^H-thymidine were 7660.7 ± 947.04, 3426.3 ± 211.84, 9207.7 ± 1267.22, 10149.5 ± 1346.50, or 482468.3 ± 18000.59 cpm, respectively (Figure 2). Irradiated splenocytes treated with 500 and 1000 μg/mL of beetroot extract increased incorporation of thymidine to about 2.69 and 2.96 times more than the control splenocytes, respectively, indicating substantially increased proliferation of the treated cells (*p* < .01 for both cases). These results suggest that beetroot extract enhanced the proliferation of the irradiated splenocytes.

### Beetroot attenuated DNA damage in splenocytes

**Figure 3.**
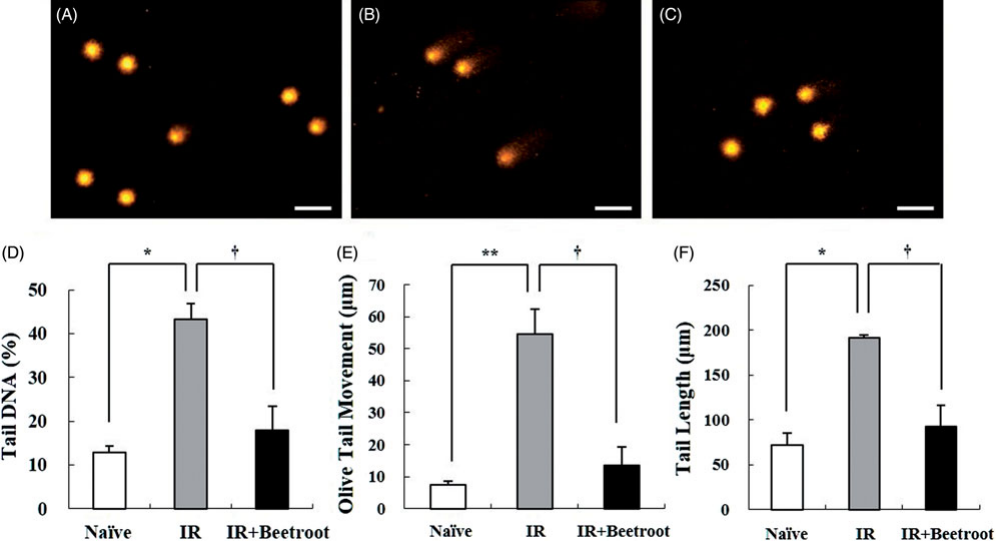
The effect of beetroot on DNA damage caused by γ-ray irradiation in splenocytes, as measured with the alkaline comet assay. Splenocytes were isolated from mice of each experiment group 10 days after 7 Gy WBI. Data shown are representative of three independent experiments. (a) Naive group, (b) IR group, (c) IR + Beetroot group, (d) percentage of tail DNA, (e) olive tail movement, (f) tail length in each group, scored according to the Komet 5.5 program. Bars in (a)–(c) represent 30 μm. Each criteria was calculated in 100 cells per mouse. Data are expressed as mean ± SEM (*, †*p <* .05, **p < .01).

Next, we looked at the influence of beetroot on DNA damage caused by WBI using comet assay. Compared with comet parameters of splenocytes of IR group, those of IR + Beetroot group were reduced significantly: they contracted by 58.7% (43.3 ± 3.5% vs. 17.9 ± 5.6%, *p* < .05) in %DNA in tail, by 75.2% (54.4 ± 7.9% vs. 13.5 ± 5.8%, *p* < .05) in olive tail movement, and by 51.8% (191.7 ± 3.1% vs. 92.4 ± 23.9%, *p <* .05) in tail length, respectively (Figure 3). This clearly indicates that beetroot inhibited DNA damage in splenocytes induced by irradiation.

### Beetroot induced proliferation of splenocytes

**Figure 4.**
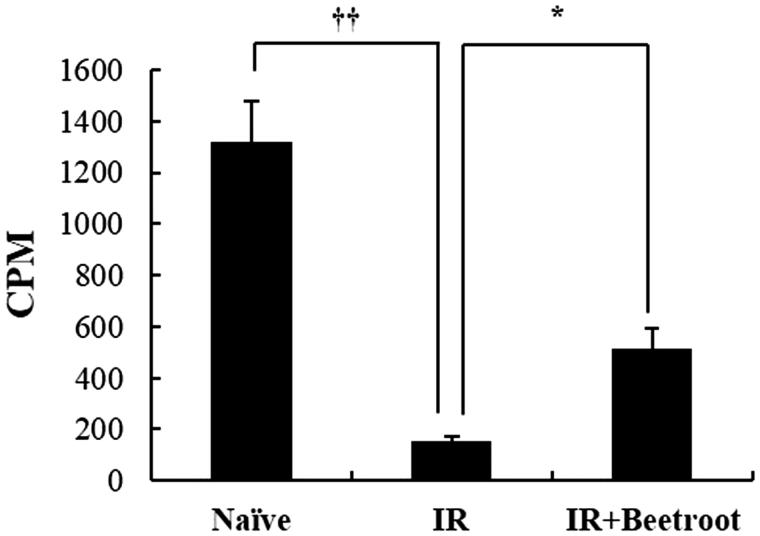
The effect of beetroot on the proliferation of splenocytes. Mice administered with beetroot extract were exposed to 7 Gy WBI and splenocytes were isolated from mice in experiment groups 10 days after irradiation. Proliferation of splenocytes was measured using the incorporation of ^3^H-thymidine. Data shown are representative of three independent experimnets and are represented as means ± SEM of radioactivity-count per minute (cpm) (*p < .05, ††*p <* .01).

To determine whether beetroot actually stimulated the proliferation of splenocytes in IR + Beetroot group, we performed the ^3^H-thymidine incorporation assay. The proliferative values of IR + Beetroot group and IR groups were 511 ± 84.5 cpm and 152.7 ± 20.7 cpm, respectively, indicating significantly increased thymidine incorporation and splenocytes count in IR + Beetroot group compare to IR group (Figure 4, *p* < .05). Proliferation level of Con-A-treated splenocytes, the positive control, was 231115.3 ± 169275.25 cpm. This result suggests that beetroot not only protected radiosensitive immune cells such as splenocytes against irradiation damage, but also stimulated their proliferation.

### Beetroot protected bone marrow cellularity in irradiated mice

**Figure 5.**
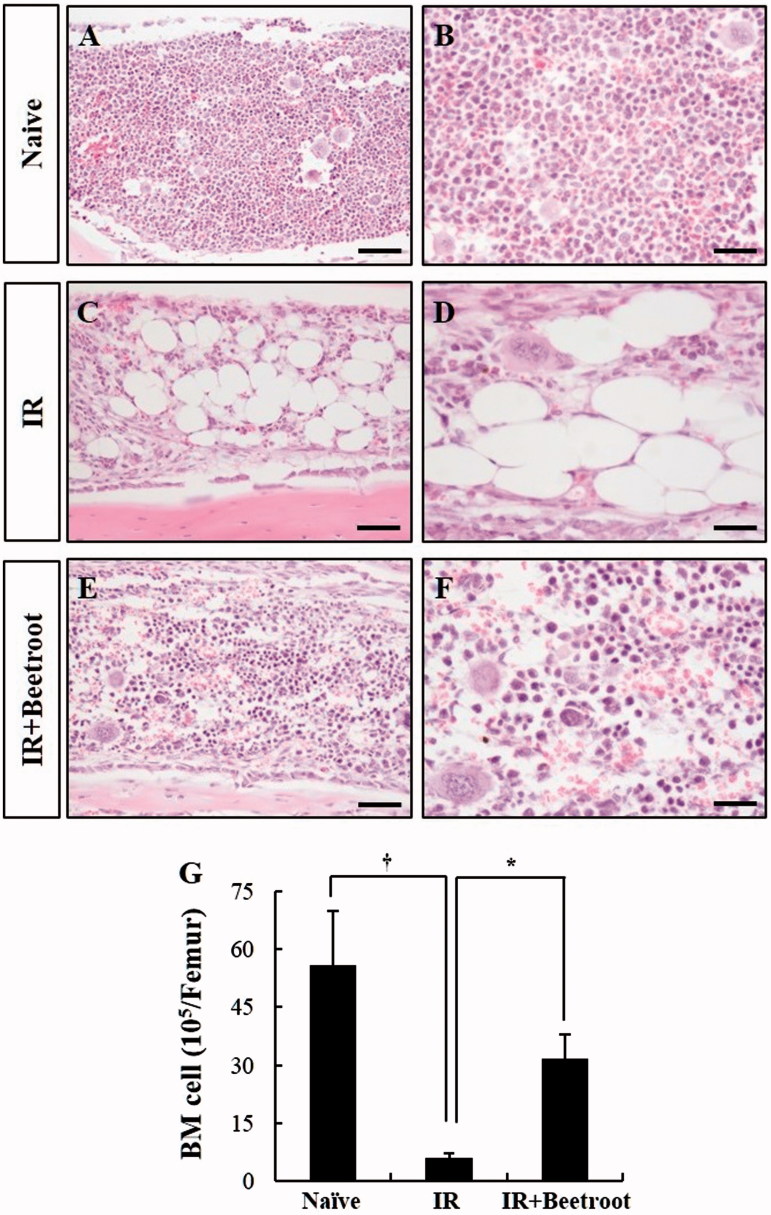
The effect of beetroot on the depletion of bone marrow cells induced by 7 Gy irradiation. (a–f) Representative images of H&E stained longitudinal sections from the femurs of (a, b) Naive, (c, d) IR and (e, f) IR + Beetroot groups on day 10 after WBI are shown. Bars =100 μm (a, c, e) and 50 μm (b, d, f). (g) Bone marrow cells were isolated from both femurs of mice of each experimental group 10 days after irradiation and counted in a haemacytometer under an optical microscope. Data are represented as means ± SEM of three independent experiments (*, †*p <* .05).

Subsequently, we assessed beetroot’s potential to protect bone marrow cells from damage induced by γ-ray irradiation. First, H&E staining was performed to evaluate the effect of beetroot on hematopoietic tissue in irradiated mice. The marrow cellularity of IR group (Figure 5(c,d)) was decreased after sublethal irradiation (7 Gy) compared with the naive group (Figure 5(a,b)). However, beetroot treatment substantially ameliorated the cellular contents of bone marrow (Figure 5(e,f)). It has been reported that a continuous increase in the number of adipocytes was observed after irradiation (Zou et al. 2016). In this study, the adipocyte area in the bone marrow of IR group increased compared with the naive group, whereas beetroot treatment decreased marrow adiposity after sublethal irradiation (Figure 5(b,d,f)). Furthermore, total number of bone marrow cells in IR + Beetroot group was significantly increased consistent with the results of H&E staining. In the IR group, bone marrow cell count was decreased by 10.9% of naive group values (*p <* .05). However, that of IR + Beetroot group was increased by 5.2 times than that of IR group (Figure 5(g), *p <* .05).

### Beetroot appeared to protect bone marrow cells against irradiation-induced apoptosis and stimulate proliferation of bone marrow cells

**Figure 6.**
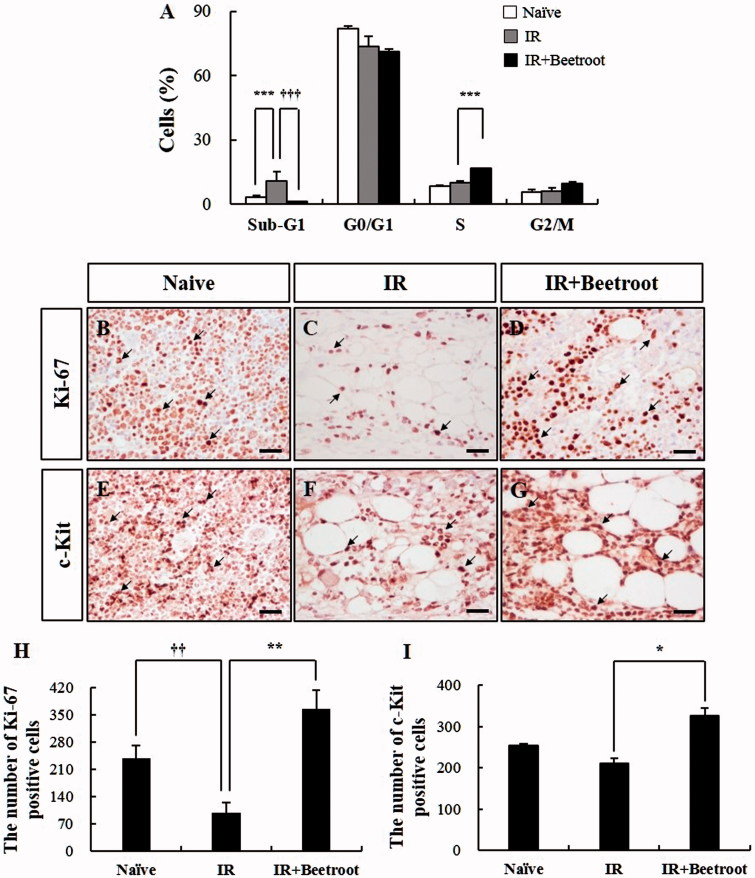
The effect of beetroot on the cells committed to hematopoiesis of bone marrow in irradiated mice. Mice administered with beetroot extract were exposed to 7 Gy irradiation and bone marrow cells of femurs were isolated from mice of each experiment group 10 days after irradiation. (a) Cell cycle of bone marrow cells of irradiated mice. Fluorescence-activated cell sorting determined the portion of bone marrow cells in the sub-G1 phase (apoptotic peak) of the cell cycle. (b–d) Representative images of Ki-67 immunoperoxidase staining in the femurs of (b) Naive, (c) IR and (d) IR + Beetroot groups. Bars =50 μm. (e–g) Representative images of immunoperoxidase staining of c-kit in the femurs of (e) non-irradiated, (f) irradiated and (g) IR + Beetroot groups. Bars =50 μm. (H-I) Quantification of (h) Ki-67 and (I) c-kit positive cells performed using five lesions showing the most representative expression of each mouse (3 mice per group). Data are represented as means ± SEM of three independent experiments (*p < .05, **,††*p* < .01, ***,†††*p* < .001).

We investigated the effect of beetroot on cell cycle progression of bone marrow cells of each experiment group after WBI. In an attempt to characterize the amount of cell deaths caused by irradiation, we measured the staining of bone marrow cells of each group with propidium iodide first and noticed the presence of apoptotic peaks. We further noticed that the cell accumulation in apoptotic peaks was increased in the IR group compared to the naive group (Figure 6(a), *p* < .001). However, IR + Beetroot group showed a dramatically lower fraction of cells in apoptotic peaks compared with the IR group (Figure 6(a), *p <* .001). Interestingly, the percentage of bone marrow cells in the S-phase was significantly increased in the IR + Beetroot group with respect to IR group (Figure 6(a), *p <* .001).

In addition, to determine whether beetroot treatment stimulated bone marrow stem cells, immunoperoxidase staining of bone marrow sections was performed with Ki-67 antibody, a proliferative index of hematopoietic cells (Budke et al. 1994). The immunoreactivity of Ki-67 was mainly observed in the areas of myeloid and erythroid hematopoiesis, but not in megakaryocytes (Figure 6(b,c,d)). The expression of Ki-67 was significantly decreased in IR group compared with Naive group (Figure 6(b,c,h), *p <* .01). In contrast, Ki-67 positive cells were dramatically increased in the IR + Beetroot group compared with the IR group (Figure 6(d,h), *p <* .01). We also examined the immunoreactivity of c-kit, an important marker of hematopoietic progenitor cells in bone marrow (Peng et al. 1998), and observed that the expression of c-kit was decreased in the IR group compared with the naive group (Figure 6(e,f,i)), but significantly increased in the IR + Beetroot group (Figure 6(g,i), *p <* .05).

Taken together, beetroot treatment-inhibited irradiation induced apoptosis and stimulated proliferation of hematopoietic progenitor cells, suggesting that beetroot possesses a radio-protective capacity against WBI-induced damage.

### Beetroot stimulated proliferation of burst forming unit erythroid (BFU-E) colonies

**Figure 7.**
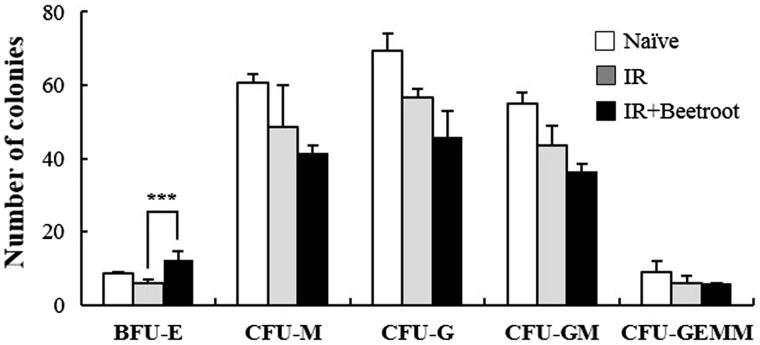
The effect of beetroot on the proliferation of bone marrow cells of irradiated mice. Bone marrow cells isolated from mice of each experimental group were cultured in methylcellulose medium for 10 days after irradiation and colonies of BFU-E, CFU-M, CFU-G, CFU-GM, CFU-GEMM were counted at 10 days after incubation. Data are shown as the mean ± SEM of three independent experiments (***p < .001).

We next investigated whether beetroot could stimulate HSCs on C57BL/6 mice under WBI (7 Gy). In the IR + Beetroot group, the measured frequency of BFU-E was significantly greater (2.1 fold) than that measured in the IR group (Figure 7, *p <* .001). However, beetroot did not alter the frequency measurements of CFU-M, CFU-G, CFU-GM, and CFU-GEMM. This result clearly indicates that beetroot stimulated the proliferation of hematopoietic progenitor cells in irradiated treatment group mice.

**Figure 8.**
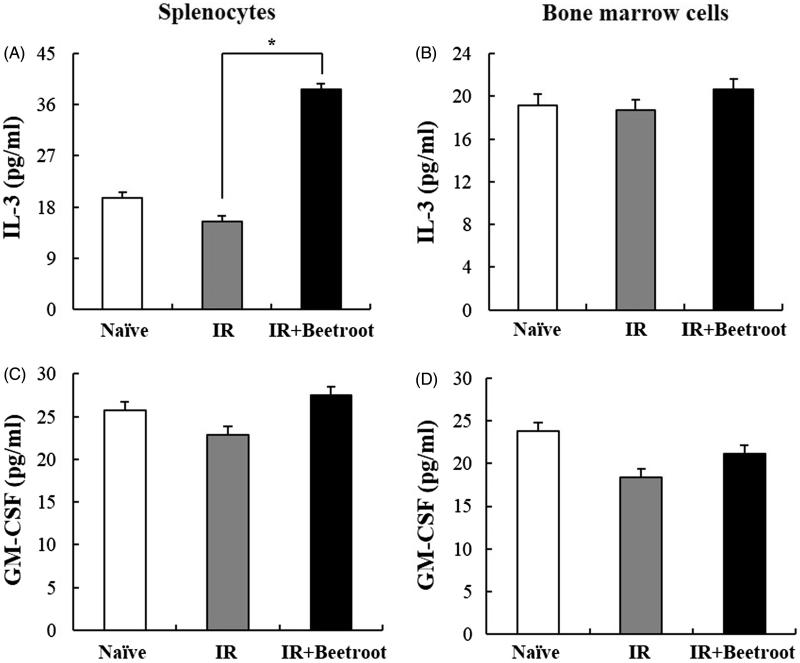
The effect of beetroot on cytokine production in splenocytes and bone marrow cells. Splenocytes and bone marrow cells were isolated from mice of each experiment group at 10 days after irradiation and seeded onto 96-well microtiter plates. After 48 h of incubation, the levels of GM-CSF and IL-3 were measured in culture supernatants. Data are represented as means ± SEM of three independent experiments. (a) IL-3 production level in splenocytes, (b) GM-CSF production level in splenocytes, (c) IL-3 production level in bone marrow cells, (d) GM-CSF production level in bone marrow cells (*p < .05).

Observing the radioprotective effects of beetroot on splenocytes via stimulating the proliferation of hematopoietic progenitor cells, we next investigated whether beetroot could stimulate growth factors or cytokines, such as GM-CSF and IL-3. The level of IL-3 was significantly elevated in splenocytes of the IR + Beetroot group compared with IR group (Figure 8(a), *p <* .05). In bone marrow cells, the level of IL-3 was also elevated in the IR + Beetroot group mice though statistically insignificance (Figure 8(b)). The level of GM-CSF showed no changes both in splenocytes and bone marrow cells of IR + Beetroot group against that of IR group (Figure 8(c–d)). These results suggest that beetroot stimulated the proliferation of hematopoietic progenitor cells in irradiated mice via inducing the secretion of cytokines associated with hematopoietic progenitor cell stimulation.

### Beetroot appeared to improve anemia induced by irradiation

**Figure 9.**
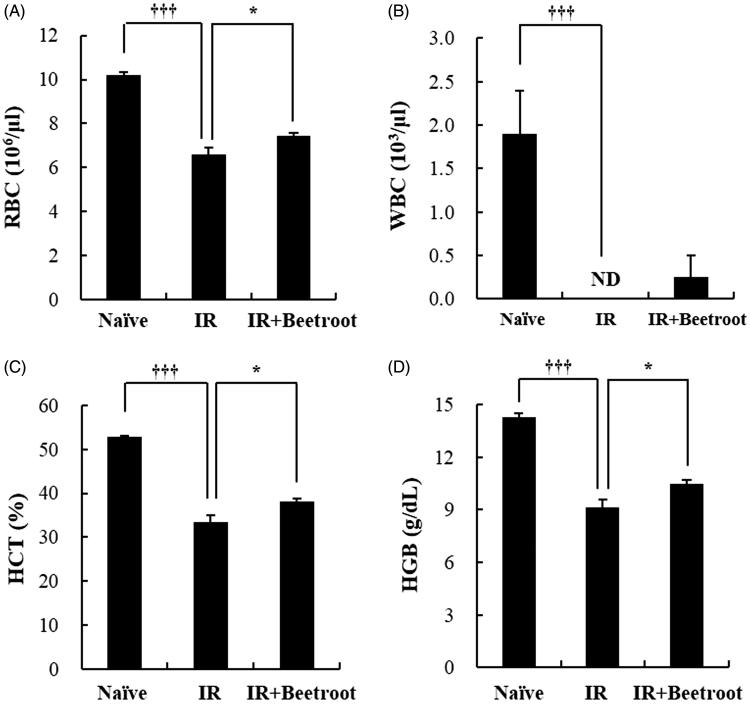
The effect of beetroot on peripheral blood in mice of each experimental group. Whole blood of each mouse was collected in EDTA-2K coated blood collection tubes and analyzed by a blood cell counter at 10 days after irradiation. Data are represented as means ± SEM of three independent experiments. (a) RBC count (10^6^/μL), (b) WBC count (10^3^/μL), (c) HCT level (%), (d) HGB level (g/dl) (*p < .05, †††*p* < .001). ND: not detected.

To explore whether beetroot exerted additional benefits on peripheral blood when mice received γ-ray irradiation, we performed the peripheral blood analysis. We observed that WBI significantly reduced the number of RBC (Figure 9(a), *p* < .001) while, in IR + Beetroot group mice, it was increased approximately ∼1.1 fold (Figure 9(a), *p <* .05). The number of WBC was significantly reduced in the IR group compared with the naive group (Figure 9(b), *p <* .001), but beetroot treatment had a tendency to elevate it in irradiated mice though it lacked statistical significance (Figure 9(b)). Beetroot treatment of irradiated mice resulted in a considerable increase in the percent of HCT (1.1 fold; Figure 9(c), *p <* .05) and the level of HGB compared with IR group mice (Figure 9(d), *p <* .05). However, no change was noted in the levels of HCV, MCH, MCHC and PLT (Data not shown). It has been reported that γ-ray irradiation could decrease the proportion of normal erythrocytes and RBCs but increase that of abnormal erythrocytes, which usually causes hemolytic anemia (Adhikari & Arora 2016). Taken together, these results indicate that beetroot could improve radiation-induced anaemia.

### Beetroot improved the survival rate of mice exposed to a lethal dose of irradiation

**Figure 10.**
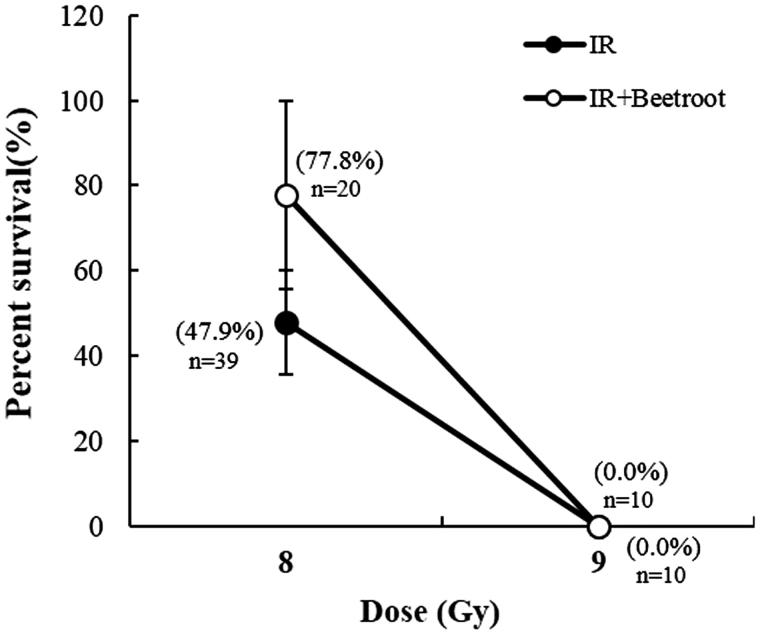
The effect of beetroot on the survival of lethally irradiated mice. Data shown are the cumulative values from three independent experiments. Numbers inserted below the percentages indicate the total number of mice in each group.

To evaluate whether beetroot could improve the survival rate of mice exposed to a lethal dose of irradiation, IR + Beetroot group were fed with the beetroot diet, a mixture of beetroot extracts and NIH-07-approved diet in the ratio of three to seven for 14 days before and after exposure to 8 or 9 Gy of WBI. The mice were observed daily for up to 30 days after irradiation. The beetroot extract treatment showed a tendency to improve the survival rate to 77.8% compared with 47.9% in IR group after a lethal irradiation of 8 Gy at the end of observation period (Table 1). In regression analysis of the survival data, the LD_50/30_ value was 8.0 Gy for IR group and 8.4 Gy for IR + Beetroot group generating a dose reduction factor (DRF) of 1.1 (Figure 10). These results demonstrate beetroot’s radioprotective effect for prolonging survival of lethally irradiated mice.

**Table 1.** The radioprotective effect of beetroot extract on lethally irradiated mice. Mice received the beetroot diet or control diet for 14 days before and after WBI, and were observed for survival during 30 days after WBI.

	Survival rate (%)	Mean survivaltime (days)
8Gy IR (*n* = 39)^a^	47.9 ± 12.2^b^	23.6 ± 1.28
8Gy IR plus Beetroot (*n* = 20)^a^	77.8 ± 22.22	26.2 ± 1.69

a Total number of mice in each group.

b Mean ± SEM of three independent experiments.

## Discussion

In this study, we confirmed the radioprotective effect of beetroot extract in splenocytes *in vitro*. Beetroot extract was not cytotoxic and stimulated the proliferation of irradiated splenocytes. Then, we have documented the radio-protective effect of beetroot on mice. First, we noted that beetroot treatment protected murine splenocytes from DNA damage. Exposure to ionizing radiation induces undesirable DNA damage, especially in highly replicating cells such as immune cells. The incurred DNA damage usually bring about apoptotic cell death and the amount of apoptosis correlates with the level of double-strand DNA breakage. Our study demonstrated that the presence of beetroot significantly reduced DNA strand breaks in splenocytes exposed to irradiation. Next, we examined whether beetroot not only protected DNA damage but also actually stimulated the proliferation of radiosensitive immune cells, and discovered that beetroot increased the proliferation capacity of irradiated splenocytes. This enhancement reflects that beetroot has the immunostimulatory potential. Taken together, we conclude that beetroot can stimulate the proliferation of irradiated splenocytes through reducing DNA damages caused by ionizing radiation.

Similar to splenocytes, bone marrow cells are also very sensitive to radiation which causes chromosomal aberrations and increases the micronucleus rates of polychromatic erythrocytes. So, we tested if beetroot treatment could protect bone marrow cells from damages induced by γ-ray irradiation. First, we compared the number of bone marrow cells in each experiment group (Naive group, IR group and IR + Beetroot group). Because irradiation can cause immediate cell death of bone marrow progenitor cells in the ensuing weeks after initial exposure leading to a hematologic crisis and eventually to hypoplasia or aplasia of bone marrow, cell counts in bone marrow can reflect its viability after an irradiation exposure (Waselenko et al. 2004). In the present study, we noted that beetroot treatment increased the marrow cellularity and total cell counts of bone marrow compared to irradiated controls after WBI. Next, we checked the amount of apoptotic cells using a flow cytometric detection method and observed a broad hypodiploid (sub-G1) peak, which can be accounted for by the partial degradation of DNA in apoptotic cells (Riccardi & Nicoletti 2006). We found that beetroot treatment not only decreased the size of the radiation-induced apoptotic cell fraction, but also increased the size of synthetic phase (S-phase) cells. In addition, immunoperoxidase staining of bone marrow sections with Ki-67 antibody, a reliable method for assessing proliferation of hematopoietic cells (Budke et al. 1994), revealed that beetroot treatment dramatically increased the expression of Ki-67 positive cells in irradiated mice. The immunoreactivity of c-kit, another important marker of hematopoietic progenitor cells in bone marrow, was also increased in the IR + Beetroot group. Taken together, we conclude that beetroot can prevent a decline in the number of bone marrow cells due to ionizing irradiation by suppressing apoptosis and by stimulating DNA synthesis and proliferation of bone marrow cells.

To further identify which progenitor cells were stimulated, we performed CFC assay taking colony formation of irradiated hematopoietic stem and progenitor cells as an indicator of sensitivity to ionizing radiation. Especially, erythroid burst forming units (BFU-E) have been reported to have the most radiation sensitive phenotype in mice, which is in line with the significant decrease of erythrocytes in peripheral blood following irradiation exposure (Imai & Nakao 1987). In the present study, beetroot stimulated the proliferation of BFU-E in the IR + Beetroot group as contrasted with the IR group and did not stimulate other colonies. So, we checked cytokines that are known to stimulate the growth of bone marrow progenitor cells and noted that the level of IL-3 secreted by beetroot treated splenic immunocytes was dramatically increased compared to that of IR group. In bone marrow cells, the level of IL-3 also tended to increase in the IR + Beetroot group bone marrow cells although it was not statistically significant. Since IL-3 stimulates the differentiation of multipotent HSCs into myeloid progenitor cells and the proliferation of all cells in the myeloid lineage in conjunction with other cytokines such as GM-CSF, a growth cytokine that stimulates stem cells to produce granulocytes and monocytes, we also checked the secretion level of GM-CSF in both splenic immunocytes and bone marrow cells. It was not altered in either splenocytes or bone marrow cells as in the result of CFC assay. Since one of the strategies of treating radiation-related bone marrow failure is to accelerate bone marrow recovery by using hematopoietic growth factors such as GM-CSF, IL-3, erythropoietin, etc. (Gale & Butturini 1990), we conclude that beetroot can stimulate erythroid burst forming units via stimulating the secretion of IL-3 in irradiated mice. When we also asked whether beetroot treatment could have an effect on peripheral blood of irradiated mice, we found that beetroot treatment led to the increase of the number of RBC, the percent of HCT and the level of HGB. This means that beetroot can also prevent anemia caused by ionizing radiation via stimulating the HSCs.

The level of granulocyte-macrophage colony-stimulating factor (GM-CSF), which has profound effects on the functional activity of various circulating leukocytes (Shi et al. 2006), was not altered as in the result of WBC count in Figure 9 where no change was noted in WBC counts between the IR group and the IR + Beetroot group. In addition, beetroot extracts did not increase the secretion level of GM-CSF and not stimulate the proliferation of CFU-M, CFU-GM, CFU-G, CFU-GEMM. It has been reported that water-soluble derivatives of propolis diminished DNA damage from ionizing radiation in WBC (Benkovic et al. 2009). However, further reports rarely proposed substances that are effective in protecting WBC against ionizing radiation although many natural or chemical substances have been studied as candidates. Here, we also conclude that beetroot mainly stimulates the factors related to RBCs rather than WBCs.

Finally, we noted that beetroot extract treatment against irradiation had beneficial effects on animal survival after WBI (DRF: 1.1). It has been reported that the survival time of lethally irradiated animals is related to various factors such as scavenging the free radicals, stimulating the hemopoietic system, and immunostimulatory activity (Arora et al. 2005). Our results of this study suggest that the radioprotective effects of beetroot on hematopoietic progenitor cells might prolong the survival time of lethally irradiated mice.

In this study, beetroot extract collected in the water fraction was used as a whole and we could not pinpoint which component of beetroot was responsible for the observed radioprotective efficacy. However, several previous studies suggest aqueous betalain pigments of beetroot as possible active ingredient. A study has shown that betalains isolated from red beets increased white blood cell count, spleen index, thymus index and activities of antioxidant enzymes (Lu et al. 2009). It has also been reported that beetroot has plenty of betalains that are related to the protection against oxidative stress and inflammation (Ninfali & Angelino 2013). Although these studies shed very little light on the protective effect of beetroot in immune cells and HSCs, its mechanism, and animal survival after WBI, considering the importance of antioxidant and anti-inflammatory activities in radioprotective mechanism, the association between betalains and radioprotection in present study seems highly plausible. Furthermore, beetroot extract not only protected splenocytes and bone marrow cells from irradiation-induced damages but also stimulated erythropoiesis via secretion of IL-3 and improved animal survival rate after lethal dose irradiation, which makes the possible association between betalains and radioprotection all the more intriguing.

## Conclusions

In summary, the administration of beetroot extracts along with γ-ray irradiation can induce significant decrease in the DNA damage and significant increase in proliferation and the stimulation of hematopoietic progenitor cells suggesting protective effects of beetroot against ionized irradiation. The aqueous extract of beetroot possesses the radio-protective effect although further studies are needed to elucidate which components of beetroot have radio-protective effects and that beetroot could be a candidate for a nontoxic radioprotective agent.
